# Digital Health Interventions and Psychosocial Outcomes in Women With Breast Cancer: Systematic Review

**DOI:** 10.2196/96858

**Published:** 2026-08-21

**Authors:** Rocío Zúñiga-Tapia, Mercè Boixadós, Eulàlia Hernández Encuentra

**Affiliations:** 1 Facultad de Enfermería, Universidad Andres Bello, Talcahuano 7100, Concepción, 4260000, Chile, +56412662318; 2 Psychology and Educational Sciences Department, Universitat Oberta de Catalunya, Barcelona, Spain

**Keywords:** breast neoplasms, quality of life, digital health, telemedicine, mobile apps, self-efficacy, mental health, anxiety, depression, systematic review

## Abstract

**Background:**

Breast cancer and its treatment can negatively affect women’s quality of life (QoL), including physical, psychological, and social well-being. Digital health interventions have been increasingly used to provide supportive care, but their effectiveness on QoL and related psychosocial outcomes remains unclear.

**Objective:**

This review aimed to evaluate the effectiveness of digital health interventions on QoL in women with breast cancer and to assess their effects on anxiety, depression, and self-efficacy.

**Methods:**

This systematic review was registered in PROSPERO (CRD420250578307) and conducted following the PRISMA (Preferred Reporting Items for Systematic Reviews and Meta-Analyses) 2020 statement. PsycINFO, Web of Science, Scopus, LILACS, and SciELO were searched in September 2025. Eligible studies were randomized controlled trials (RCTs) or controlled quasi-experimental studies with preintervention and postintervention assessments that evaluated digital health interventions in women with breast cancer during or up to 1 year after active treatment and reported QoL outcomes. Two reviewers independently screened all records (interrater agreement >90%) and extracted data using a standardized template, with independent verification by a second reviewer. Risk of bias was assessed using the Cochrane Risk of Bias tool (RoB 2) for RCTs and the Risk of Bias in Non-randomized Studies of Interventions (ROBINS-I) tool for the quasi-experimental study; findings were synthesized narratively following the Synthesis Without Meta-analysis (SWiM) guideline.

**Results:**

A total of 408 records were identified, and 15 studies were included: 14 RCTs and 1 quasi-experimental study. Sample sizes ranged from 35 to 394 participants. Ten studies evaluated internet-based approaches such as websites or apps; 3 used text messaging (eg, WhatsApp [WhatsApp LLC] or WeChat [Tencent Technology, Shenzhen Company Limited]), 1 used virtual reality, and 1 used an email listserve. The direction of effect favored the digital intervention for QoL in 8 of the 15 studies. Self-efficacy was assessed in 5 studies and favored the intervention in only 1, with the effect not maintained at follow-up. Anxiety, depression, or emotional distress were assessed in 13 studies, with a favorable direction of effect in 5. Retention rates ranged from 68.6% to 100%. Among the 14 RCTs, 3 were judged at high overall risk of bias, and 11 raised some concerns; none was at low risk. The quasi-experimental study had a serious overall risk of bias. Certainty of evidence was low for QoL and emotional outcomes and very low for self-efficacy.

**Conclusions:**

Digital health interventions may provide modest, short-term improvements in some dimensions of QoL in women with breast cancer. Favorable effects appeared more often in interventions with active therapeutic components or professional support; however, this pattern was based on indirect comparisons across heterogeneous studies. Effects on self-efficacy and mental health were inconsistent, and acceptability was insufficiently evaluated. Although promising, current evidence does not support strong or generalizable conclusions.

## Introduction

The World Health Organization (WHO) identifies breast cancer as one of the leading public health challenges globally and the most prevalent malignancy in both high-income and low- and middle-income countries [1]. According to estimates from the Global Cancer Observatory, more than 2 million cases of breast cancer were recorded worldwide in 2022, with an age-standardized rate of 46.8 per 100,000 women [2]. In addition, projections indicate that global breast cancer cases will increase by 38% by 2050, and annual deaths from this disease will rise by 68% [3].

Women with breast cancer undergoing oncological treatment experience a range of physical, psychological, and social consequences [4,5]. Fatigue, pain, lymphedema, sexual dysfunction, and sleep disturbances are frequently reported as physical sequelae of breast cancer [6-8]. Psychological consequences such as anxiety, depression, and fear of cancer recurrence have also been demonstrated, with negative impacts on health and quality of life (QoL) [9,10]. In this context, psycho-oncological interventions have shown efficacy in improving coping capacity, enhancing self-efficacy skills, and reducing stress and emotional distress [11-13].

In light of the foregoing, digital health—defined by WHO [14] as the “field of knowledge and practice associated with the development and use of digital technologies to improve health, encompassing other uses of digital technologies for health such as the Internet of Things, advanced computing, big-data analytics, artificial intelligence, including machine learning and robotics”—emerges as a platform through which diverse interventions can be deployed. Before the COVID-19 pandemic, digital health had not been widely incorporated into standard oncological care worldwide [15]; however, its use has increased substantially in recent years. Systematic reviews have examined the impact of digital health among patients with cancer, revealing that digital interventions—such as informational websites including patient narratives, mindfulness intervention apps, physical activity programs, virtual reality (VR) for pain management, and videoconferencing—produce significantly positive outcomes in patient-reported symptoms, fatigue and pain levels, health-related QoL, functional capacity, and depression levels compared with care delivered without such tools [16-18].

AI and other advanced digital technologies are increasingly being applied across the breast cancer care continuum, including screening, diagnostic support, risk stratification, treatment planning, symptom monitoring, and survivorship support [19]. However, this review focuses specifically on digital health interventions directed at supportive care and psychosocial outcomes rather than diagnostic or treatment-decision technologies.

Given the growing use of digital health interventions in oncology, and the heterogeneity in the available findings, this systematic review aimed to evaluate the effectiveness of digital health interventions on QoL in women with breast cancer, and to assess their effects on self-efficacy and mental health outcomes.

## Methods

### Overview

This systematic review was registered in PROSPERO (CRD420250578307) [20] and its protocol was previously published [21]. It was performed in accordance with the PRISMA (Preferred Reporting Items for Systematic Reviews and Meta-Analyses) guidelines [22] and following the Population, Intervention, Comparison, Outcomes, and Context (PICOC) [23]. This review addressed the following question (PICOC): in women with breast cancer during active treatment or up to 1 year after completion of active treatment (Population), do digital health interventions (Intervention), compared with usual care, standard treatment, or a waitlist (Comparator), improve QoL and, secondarily, self-efficacy, anxiety, and depression (Outcomes), and eligible interventions were primarily directed at women with breast cancer, irrespective of geographic location or care setting (Context)?

The following amendments to the registered protocol, made during the conduct of the review, are reported in accordance with PRISMA 2020 (item 24c) as detailed in Checklist 1. First, it is specified that the study designs correspond to both randomized and nonrandomized studies (randomized controlled trials [RCTs] and controlled quasi-experimental studies with preintervention and postintervention assessments). Second, Scopus was searched instead of MEDLINE, representing a deviation from the registered protocol; this was complemented by hand-searching the reference lists of the included studies. Third, risk of bias was assessed according to study design, using the revised Cochrane Risk of Bias tool (RoB 2) [24] for RCTs and the Risk of Bias in Non-randomized Studies of Interventions (ROBINS-I) tool [25] for the quasi-experimental study, in accordance with the amended protocol. Fourth, the certainty of the randomized evidence was rated using the GRADE (Grading of Recommendations Assessment, Development and Evaluation) approach [26,27], an assessment not specified in the protocol, to inform confidence in the findings. Fifth, eligibility was restricted to women from diagnosis through active treatment and up to 1 year after completion of active treatment; studies of long-term survivors (more than 1 year after completing active treatment) were excluded, a temporal restriction of the population that was not specified in the registered protocol. In addition, references were managed in Rayyan [28] (Rayyan Systems Inc; rather than the registered Mendeley) and PsycINFO was searched through the ProQuest platform (ProQuest LLC; rather than EBSCOhost); these operational changes did not affect the eligibility criteria or the synthesis.

### Search Strategy

The search was conducted in September 2025 using PsycINFO (ProQuest), Web of Science, Scopus, LILACS, and SciELO. Database-specific free-text terms were adapted to the syntax and indexing fields of each database. The complete search strategies are provided in Multimedia Appendix 1. All articles retrieved from the databases were exported to Rayyan software for screening [28]. The auto-resolve function was used, and any records with less than 95% similarity were manually reviewed by the lead researcher (RZ-T).

Title and abstract screening were conducted independently and in duplicate by 2 reviewers (RZ-T and MB); articles that did not meet the criteria were removed, and those with insufficient information to make a decision were carried forward to the full-text screening stage.

The full text of all potentially eligible records was likewise assessed independently and in duplicate by the same 2 reviewers. Interrater agreement was high (>90%) [29], and any disagreements were resolved through discussion or, when needed, with a third reviewer (EHE).

### Eligibility Criteria

#### Overview

In this review, digital health interventions were defined as the use of digital technologies—such as computers, mobile devices, wearables, and the internet—to deliver or support health-related information, services, or care. Because this is not a uniform category, interventions were interpreted according to their function: informational patient portals or websites, moderated peer-support forums, symptom monitoring and remote tracking, telerehabilitation, digitally delivered psychological therapy (eg, cognitive behavioral therapy [CBT] or mindfulness), and VR.

#### Inclusion Criteria

The inclusion criteria were as follows:

1. Articles in English, Spanish, or Portuguese.
2. Studies (papers or theses in databases) with research designs: RCTs and controlled quasi-experimental studies with preintervention and postintervention assessments.
3. Women with breast cancer, from diagnosis through active treatment and up to 1 year after completion of active treatment.
4. Digital interventions had to deliver or support a health-related component through a digital device or platform and report at least one dimension of health-related QoL as an outcome (eg, mindfulness, exercise, and self-efficacy activities).
5. Outcomes must include any dimension of health-related QoL (eg, physical, social, psychological, and symptom dimensions).
6. Studies must provide details about the study design, sample, and intervention components that allow the analysis to be carried out.

#### Exclusion Criteria

The exclusion criteria were as follows:

1. Nonempirical studies (eg, theoretical or conceptual articles)
2. Reviews, editorials, letters, expert opinions, protocols, or preprints
3. Studies focusing on long-term survivors (more than 1 year after completing active treatment), patients in palliative or end-of-life care, or male patients.

### Data Extraction

Data were extracted by the lead author (RZ-T) using a Microsoft Excel template designed to collect details about each digital health intervention and were independently verified by a second reviewer (MB) for all included studies, with any discrepancies resolved by consensus. The data included (1) study characteristics (country, design, sample size, target population, recruitment setting, objective, and methods), (2) primary outcomes of the digital health intervention (mean change and effect size, if applicable), and (3) secondary outcomes of the digital health intervention (mean change and effect size, if applicable).

### Quality Assessment

The risk of bias of each included study was assessed according to its design. RCTs were assessed using the revised Cochrane RoB 2 tool for randomized trials [24], which evaluates 5 domains—the randomization process, deviations from the intended interventions, missing outcome data, measurement of the outcome, and selection of the reported result—through signaling questions, yielding a domain-level judgment and an overall judgment of “low risk,” “some concerns,” or “high risk.” The nonrandomized quasi-experimental study was evaluated using the ROBINS-I tool [25], as specified in the amended protocol. ROBINS-I assesses bias due to confounding, selection of participants, classification of interventions, deviations from intended interventions, missing data, measurement of outcomes, and selection of the reported result. Each study was assessed independently by 2 reviewers (RZ-T and MB), and disagreements were resolved by consensus or with a third reviewer (EHE).

### Data Synthesis

Meta-analysis was not undertaken because the included studies showed substantial clinical and methodological heterogeneity in intervention type and content, comparator conditions, outcome constructs and measurement instruments, and assessment time points. In addition, comparable numerical effect estimates and measures of precision were not consistently reported across studies. Therefore, the findings were synthesized narratively in accordance with the Synthesis Without Meta-analysis (SWiM) reporting guideline [30] and Cochrane guidance for synthesizing findings using methods other than meta-analysis [31]. Studies were grouped according to the outcome domain—QoL (primary) and, secondarily, self-efficacy, anxiety, and depression—and, within each domain, by intervention function (informational portals or websites, moderated peer-support forums, symptom monitoring, telerehabilitation, digitally delivered psychological therapy, and VR), level of human support (guided vs unguided), and length of follow-up. The direction of effect was determined primarily from between-group comparisons at the principal postintervention assessment reported in each study. Findings were classified as favoring the digital intervention, favoring the comparator, or showing no clear direction of effect. Statistical significance was reported when available but was not used as the sole criterion for determining the direction of effect. The certainty of the evidence for each outcome was rated using the GRADE approach [26,27]. The quasi-experimental study was included in the narrative synthesis but was not incorporated into the GRADE certainty assessments.

## Results

### Study Selection

**Figure 1.**
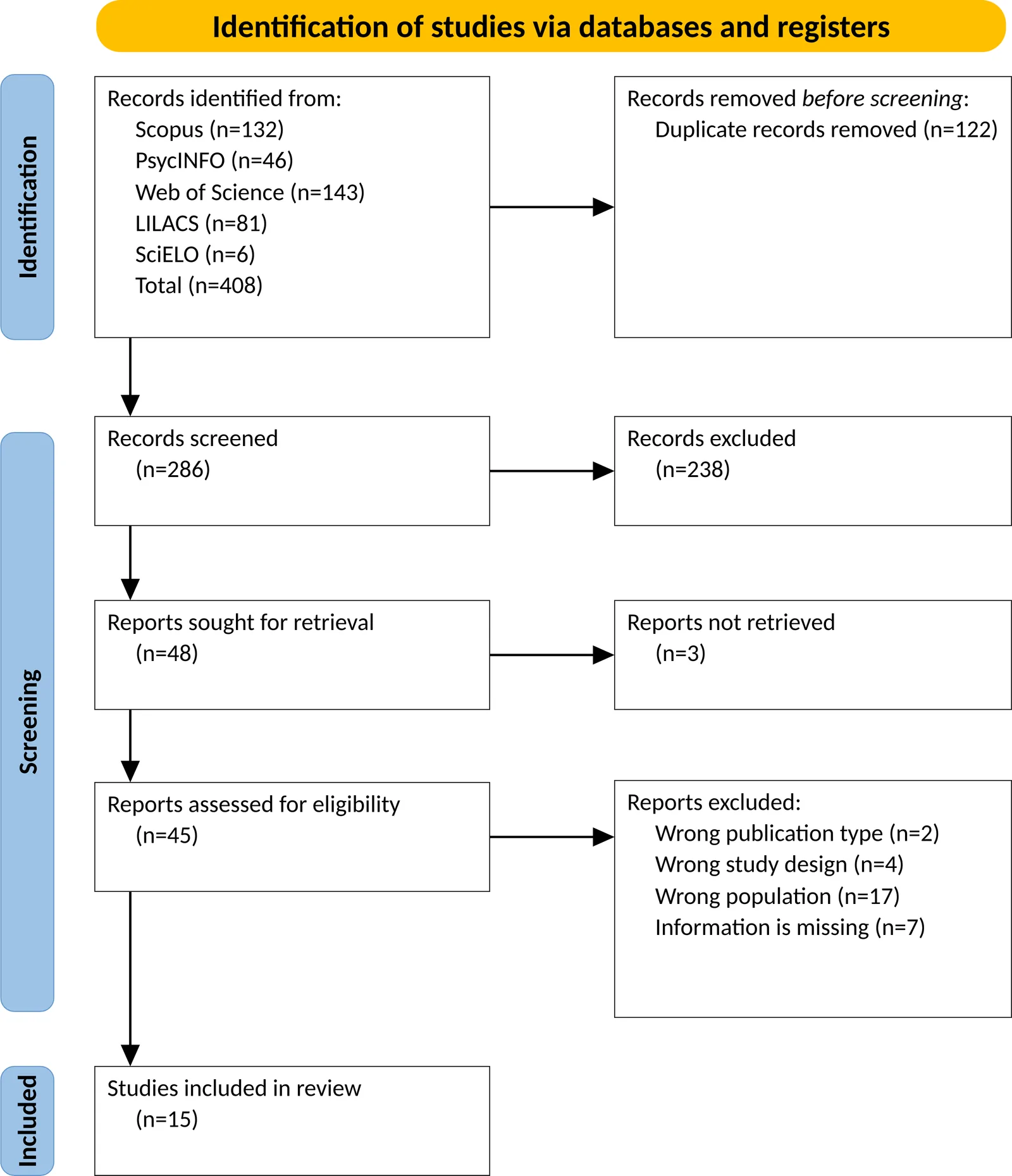
PRISMA (Preferred Reporting Items for Systematic Reviews and Meta-Analyses) 2020 flow diagram for new systematic reviews, which included searches of databases and registers only.

A total of 408 records were identified from 5 databases and uploaded to Rayyan. After removal of 122 duplicate records, 286 records underwent title and abstract screening, of which 238 were excluded. Forty-eight reports were sought for retrieval; 3 could not be retrieved, and 45 full-text reports were assessed for eligibility. Thirty reports were excluded: 2 because of publication type, 4 because of study design, 17 because of population, and 7 because insufficient information was available to confirm eligibility. Fifteen studies met the eligibility criteria and were included in the review [32-46]. The study selection process is presented in Figure 1, and study-specific reasons for full-text exclusion are provided in Multimedia Appendix 2.

### Study Characteristics

Table 1 includes a summary of the participant characteristics of the included studies. The 15 included studies [32-46] had a sample size ranging from 35 to 394, with studies being conducted in China (4/15, 26.7%) [35-37,46], Australia (3/15, 20%) [34,38,43], the United States (2/15, 13.3%) [32,41], and Turkey (2/15, 13.3%) [39,44], and one study each in Finland [33], Japan [42], Brazil [40], and Singapore (1/15, 6.7% each) [45], and were published between 2010 and 2025. Participants were adult women, with mean ages 43.6 (SD 5.0) to 63.9 (SD 7.8) years. In terms of research methodology, 14 studies were RCTs [32-45], and 1 was a quasi-experimental study with pre- and postintervention assessments [46].

**Table 1.** Participant characteristics of the included studies.

Author and year	Country (design)	Sample size, randomized, enrolled, and analyzed, n (IG^a^:CG^b^)	Age (years), IG:CG, mean (SD)	Breast cancer stage	Breast cancer therapy
Salzer et al (2010) [32]	The United States (RCT^c^)	78 (51:27)	Not reported	I, II	Surgery, chemotherapy, radiotherapy, endocrine therapy
Ryhänen et al (2013) [33]	Finland (RCT)	98 (50:48)	54.4 (40-67):55.7 (41-69)^d^	I, II, III, IV	Surgery, chemotherapy, radiotherapy, endocrine therapy, targeted therapy
White et al (2018) [34]	Australia (RCT)	394 (212:182)	43.6 (5.0):43.9 (5.3)	I, II, III	Surgery, chemotherapy, radiotherapy, endocrine therapy, targeted therapy
Zhu et al (2018) [35]	China (RCT)	114 (57:57)	46.2 (8.5):48.2 (8.1)	I, II, III, IV	Surgery, chemotherapy
Wang et al (2022) [36]	China (RCT)	103 (51:52)	45.37 (7.59):48.17 (8.05)	0, I, II, III, IV	Surgery, chemotherapy, radiotherapy, immunotherapy
Zhang et al (2022) [37]	China (RCT)	98^e^; 90^f^ (45:45)	52.29 (7.69):51.03 (7.98)	I, II, III, IV	Chemotherapy
Singleton et al (2023) [38]	Australia (RCT)	Randomized: 160 (80:80) Included in the principal analysis: 156 (78:78)	53.8 (9.6):55.7 (12.1)	I, II, III	Surgery, chemotherapy, radiotherapy, endocrine therapy, targeted therapy
Bahçaci et al (2024) [39]	Turkey (RCT)	64 (33:31)	49.1 (8.8):44.7 (10.0)	I, II, III, IV	Surgery, chemotherapy
de Aviz et al (2024) [40]	Brazil (RCT)	48 (24:24)	50.7 (11.6):49.3 (10.1)	I, II, III	Surgery, chemotherapy, radiotherapy, endocrine therapy
Graetz et al (2024) [41]	The United States (RCT)	304 (200:104)^g^	58.7 (10.5):58.3 (11.4)^g^	0, I, II, III	Chemotherapy, radiotherapy
Okuyama et al (2024) [42]	Japan (RCT)	Randomized: 130 (65:65) Analyzed: 125 (61:64)	63.9 (7.8):62.7 (7.4)	I, II, III	Surgery, chemotherapy, radiotherapy, endocrine therapy
Rigg et al (2024) [43]	Australia (RCT)	35 (17:18)	58.42 (10.88):56.73 (8.44)	IV	Surgery, chemotherapy, radiotherapy
Salman Saraç et al (2024) [44]	Turkey (RCT)	88 (44:44)	48.8 (9.0):49.3 (9.4)	Not reported	Surgery
Pang et al (2025) [45]	Singapore (RCT)	123 (62:61)	56.6 (11.7):55.4 (10.9)	0, I, II, III	Surgery
Geng et al (2024) [46]	China (quasi-experimental study)	60 (30:30)	52.50 (45.75-59.00):57.50 (44.50-62.25)^h^	I, II, III, IV	Surgery, chemotherapy

a IG: intervention group.

b CG: control group.

c RCT: randomized controlled trial.

d range, not SD.

e Recruited.

f Analyzed.

g Used a 3-arm design (IG1, n=98: mean age 59.4, SD 10.3; IG2, n=102: mean age 58.1, SD 10.7).

h median (IQR), not mean (SD).

### Treatment Conditions

A total of 10 (66.7%) studies primarily evaluated internet-based interventions, including websites or mobile apps for patients with breast cancer [33-35,40-46]. One of these studies combined an application with tailored SMS support [46]. The remaining 3 (20%) studies used SMS text messages through different apps such as SMS, WeChat, WhatsApp, or others [36,38,39]. One study used head-mounted VR glasses [37], and one study used an email listserve [32].

The intervention methods included 6 (40%) studies that used psychological intervention strategies as the core component, including online peer support programs [32], mindfulness [36], stress reduction techniques through VR [37], and an online CBT-based psychosocial program [43], along with 2 studies focused on self-efficacy [35,45]. Regarding educational strategies, they were primarily included in 2 (13.3%) studies, providing cancer-related information, symptom management education, and emotional support [34,44]. Two studies worked with empowerment strategies [33,38] (13.3%), 2 studies involved remote symptom monitoring [41,42], and 3 studies focused on physical exercises [39,40,46]. The duration of these interventions ranged from 29 days to 12 months, depending on the study. The professionals involved in these interventions were nurses, psychologists, doctors (physicians or oncologists), physiotherapists, among others. Table 2 summarizes the intervention characteristics of the included studies.

**Table 2.** Summary of study characteristics.

Author and year	Intervention group	Contents	Control group	Intervention duration (data collection points)	Instrument of measurement	Results
Salzer et al (2010) [32]	Unmoderated, closed internet listserve (peer-to-peer group)	Support and emotional experiences shared by participants without professional structure	Review of information on a cancer-related website	12 months (baseline, 4 months, and 12 months)	FACT-B^a^ SESES-C^b^ HSCL-25^c^ IES^d^ POMS^e^ HHI^f^ MOS-SSS^g^	No significant differences in QoL^h^, self-efficacy, hope, cancer-specific stress, and social support between groups Psychological distress and mood improved in CG^i^
Ryhänen et al (2013) [33]	Internet-based breast cancer patient pathway portal	Education program which includes flow chart diagram of the patient pathway during breast cancer treatment, from diagnosis through surgery, chemotherapy, and follow-up	Usual care	9 months (baseline, surgery, after surgery, meeting with oncologist, before and after chemotherapy, before and after radiotherapy, and 12 months postdiagnosis)	QoL Scale Breast Cancer^j^ STAI^k^ Own questionnaire of side effects	No significant differences in QoL, anxiety, or side effects between groups. Anxiety decreased in both groups
White et al (2018) [34]	“Information for Me” website	Information resources on diagnosis, surgery, chemotherapy, radiotherapy, and survivorship, emotional responses, and support services	Usual care	6 months (baseline, 3 months, and 6 months)	FACT-B HADS^l^ SCNS-BC^m^	General QoL showed an improvement in the IG^n^ than the CG at 3 months, but not at 6 months No significant differences between groups in anxiety, depression, QoL specific, and unmet needs
Zhu et al (2018) [35]	Breast cancer e-Support app	Learning, discussion, ask-the-expert, and personal stories forums moderated by health care professionals	Usual care	3 months (baseline, 3 months, and 6 months follow-up)	FACT-B SICPA^o^ HADS MDASI^p^ MSPSS^q^	At 3 months, QoL, self-efficacy, and social support improved in IG At 6 months, no significant differences were observed between the groups
Wang et al (2022) [36]	Internet-delivered mindfulness-based cancer recovery program delivered through WeChat	Mindfulness techniques including body scan, mindful breathing, self-compassion, integrating mindfulness into daily life, materials, and audios provided	Usual care with health education on managing cancer symptoms, therapy side effects, diet, and exercise	1 month (baseline, postintervention, and 1-month follow-up)	FACT-B MDASI	Significant improvements in QoL and symptom burden in the IG compared to the CG. Emotional distress decreased only at postintervention, but not at 1 month follow-up
Zhang et al (2022) [37]	VR-CALM^r^ virtual reality–based psychotherapy intervention for symptom management	Psychological support (symptom management, self, relationships, meaning, spirituality, and future hope)	Usual care	3 months (baseline and 3 months)	FACT-B DT^s^ SAS^t^ SDS^u^ CARS^v^ PFS^w^ PSQI^x^	QoL, distress, anxiety, depression, fatigue, and sleep outcomes favored the VR-CALM group after the intervention
Singleton et al (2023) [38]	EMPOWER-SMS text messages delivered	Physical activity, healthy diet, social and emotional well-being, endocrine therapy adherence, and side-effects management	Usual care plus 1 welcome SMS and 1 follow-up interview reminder SMS	6 months (baseline and 6 months)	EORTC QLQ-C30^y^ or EORTC QLQ-BR23^z^ SEMCD^aa^ DASS-21^ab^ GPAQ^ac^ Own self-reported endocrine therapy adherence BIPQ^ad^ BMI	No clear between-group differences were observed for QoL, self-efficacy, depression, anxiety, stress, physical activity, BMI, or body composition. Therapy adherence improved in IG
Bahçaci et al (2024) [39]	Tele-rehabilitation-based relaxation exercises delivered via WhatsApp	Jacobson’s progressive relaxation focusing on muscle relaxation with breathing exercises	Relaxation exercise brochure	1.5 months (baseline and 1.5 months)	EORTC QLQ-C30 BPI^ae^ HADS FIS^af^ FACT-Cog^ag^ PSQI	IG showed a significant improvement in QoL (symptom scale), depression, anxiety, sleep quality, and cognitive function. Fatigue and pain decreased in IG
de Aviz et al (2024) [40]	Asynchronous telerehabilitation via messaging app	Exercise videos focusing on shoulder mobility, strength, and aerobic activity	Usual care	1.5 months (baseline, day 15, and 1.5 months)	FACT-G^ah^ FACIT-F^ai^ VAS^aj^	QoL showed significant enhancement in IG Fatigue and pain were significantly reduced in the IG
Graetz et al (2024) [41]	Mobile app	App: symptom and medication adherence monitoring, with weekly symptom reporting and alerts to the oncology team App and feedback weekly tailored text messages	Usual care	6 months (baseline and 12 months)	SF-12^ak^ PROMIS SEMS^al^ FACT-ES^am^ CPPPA^an^ Own self-reported adherence therapy	QoL, self-efficacy, and symptom burden with no significant differences between groups App and feedback group showed decreases in total and high-cost health care encounters and office visits
Okuyama et al (2024) [42]	ePRO monitoring app	PRO-CTCAE symptoms (insomnia, joint pain, headache, anxiety, and hot flashes) plus daily status/medication; staff followed up as needed	Usual care	3 months (baseline and 3 months)	FACT-B / B-TOI ^ao^ EORTC QLQ-COMU26^ap^ PRO-CTCAE	No significant differences in QoL and communication between health providers High ePRO response maintained to week 10
Rigg et al (2024) [43]	Finding My Way-Advanced: self-directed online CBT-based program (website)	Managing fear of progression, emotional distress, and physical symptoms with role functioning and social support	Usual care	1.5 months (baseline and 1.5 months)	QLQ-C30 DASS-21 FOP^aq^ SCNS - Short Form PTSD Symptom Scale-Self Report^ar^ SCNS	QoL and role functioning, cancer-specific distress, and fear of progression showed improvements in the IG Mental QoL, general distress, and social functioning deteriorated in the IG vs CG
Salman Saraç et al (2024) [44]	“Breast Cancer Surgery Information Guide” informative mobile app	Surgery, treatment, and recovery information. It contained videos, text, and reminders	Routine clinic care/training only; no app	1 month (baseline and 3 weeks after surgery)	FACT-G HAD-A only anxiety NCCN-DTPL^as^	QoL showed no significant differences between groups. Anxiety and distress showed significant reduction in IG
Pang et al (2025) [45]	iCareBreast mobile app	Perioperative care guidance, breast cancer education, psychological support, and social support	Usual care with educational pamphlets	29 days around surgery; measures at T0, T1, T2 (baseline, 2 weeks post surgery, and 2.5 months follow-up)	EORTC QLQ-BR23 GSES^at^ HADS MFSI^au^ Own Perioperative Care Satisfaction Scale.	No clear between-group differences were observed for QoL, self-efficacy, anxiety, depression, and fatigue at follow-up. Satisfaction was higher in the IG at the final assessment, but the between-group difference was not statistically significant
Geng et al (2024) [46]	“Breast Care” app combined with tailored SMS-based physical activity support	Physical activity intervention based on social cognitive theory, self-efficacy theory, and the theory of planned behavior, with personalized goals, self-monitoring, feedback, tailored messages, and support	Usual care plus printed educational material, face-to-face health education during chemotherapy visits	3 months (baseline and postintervention assessment at 3 months)	FACT-B HADS; IPAQ-SF^av^	At 3 months, QoL and anxiety favored the IG, mainly because outcomes deteriorated in the CG; no significant within-group improvement was observed in the IG

a FACT-B: Functional Assessment of Cancer Therapy – Breast.

b SESES-C: Stanford Emotional Self-Efficacy Scale – Cancer.

c HSCL-25: Hopkins Symptom Checklist-25.

d IES: Impact of Event Scale.

e POMS: Profile of Mood States.

f HHI: Herth Hope Index.

g MOS-SSS: Medical Outcomes Study-Social Support Survey.

h QoL: quality of life.

i CG: control group.

j QoL Breast Cancer: Quality of Life Instrument - Breast Cancer Patient Version.

k STAI: The State-Trait Anxiety Inventory.

l HADS: Hospital Anxiety and Depression Scale.

m SCNS-BC: Supportive Care Needs Survey-Breast Cancer.

n IG: intervention group.

o SICPA: Stanford Inventory of Cancer Patient Adjustment.

p MDASI: MD Anderson Symptom Inventory.

q MSPSS: Multidimensional Scale of Perceived Social Support.

r VR-CALM: Virtual Reality Comprehensive Assisted Learning and Management.

s DT: distress thermometer.

t SAS: Self-Rating Anxiety Scale.

u SDS: Self-Rating Depression Scale.

v CARS: Concerns about Recurrence.

w PFS: Piper Fatigue Scale.

x PSQI: Pittsburgh Sleep Quality Index.

y EORTC QLQ-C30: European Organisation for Research and Treatment of Cancer Quality of Life Questionnaire –Core 30.

z EORTC QLQ-BR23: European Organisation for Research and Treatment of Cancer Quality of Life Questionnaire–Breast Cancer Module 23.

aa SEMCD: Self-Efficacy for Managing Chronic Disease Scale.

ab DASS-21: Depression Anxiety and Stress Scale.

ac GPAQ: Global Physical Activity Questionnaire.

ad BIPQ: Brief Illness Perception Questionnaire.

ae BPI: Brief Pain Inventory.

af FIS: Fatigue Impact Scale.

ag FACT-Cog: Functional Assessment of Cancer Therapy – Cognitive Function.

ah FACT-G: Functional Assessment of Cancer Therapy – General.

ai FACIT-F: Functional Assessment of Chronic Illness Therapy – Fatigue.

aj VAS: Visual Analogue Scale.

ak SF-12: Short Form 12-Item Health Survey (Physical and Mental Component Summary).

al PROMIS SEMS: Patient-Reported Outcomes Measurement Information System Self-Efficacy for Managing Symptoms.

am FACT-ES: Functional Assessment of Cancer Therapy - Endocrine Symptoms.

an CPPPA: 10-item Communication: Patient and Physician Peer Assessment.

ao FACT-B /B-TOI: FACT-B Trial Outcome Index.

ap EORTC QLQ-COMU26: European Organization for Research and Treatment of Cancer Quality of Life Questionnaire-Communication 26.

aq FOP: Fear of Progression Questionnaire.

ar PTSD Scale-Self Report: Post-Traumatic Stress Scale-Self Report.

as NCCN- DTPL: NCCN Distress Thermometer and Problem List.

at GSES: General Self-Efficacy Scale.

au MFSI: Multidimensional Fatigue Symptom Inventory.

av IPAQ-SF: International Physical Activity Questionnaire–Short Form.

### Primary Outcome

The reviewed articles all delivered interventions that had the goal, either primary or secondary, of improving QoL, consistent with the inclusion criteria. All included studies assessed QoL as either a primary or secondary outcome, in accordance with the eligibility criteria. The studies used both breast cancer–specific and generic validated QoL instruments. FACT instruments were used in 9 (60%) studies [32,34-37,40,42,44,46], European Organisation for Research and Treatment of Cancer Quality of Questionnaire–Core 30 (EORTC QLQ-C30) or European Organisation for Research and Treatment of Cancer Quality of Life Questionnaire–Breast Cancer Module 23 (EORTC QLQ-BR23) instruments in 4 (26.7%) studies [38,39,43,45], the Quality-of-Life Instrument–Breast Cancer Patient Version in 1 study [33], and the Short Form 12-Item Health Survey (SF-12) in 1 study [41]. Where studies used multiple QoL-related instruments, classification was based on the measure covering the broadest range of QoL dimensions; therefore, the single-domain FACT-Cog [39] and FACT-ES [41] subscales were excluded from the FACT tally to avoid double-counting. Following the SWiM approach, findings were summarized by the direction of effect rather than by statistical significance alone. Seven [34-37,39,40,43] of the 14 RCTs [32-45] showed a favorable direction of effect, whereas the remaining RCTs showed no clear between-group benefit or mixed findings [32,33,38,41,42,44,45]. The controlled quasi-experimental study also favored the intervention group, although this primarily reflected less deterioration relative to the control group rather than a clear within-group improvement [46]. Favorable effects appeared more frequently among interventions containing active therapeutic, behavioral, or rehabilitation components. However, because intervention formats, populations, and comparators differed substantially, this pattern should be regarded as exploratory rather than evidence of a moderating effect. Overall, the randomized evidence suggested a possible short-term benefit for QoL, with low certainty.

### Other Outcomes: Self-Efficacy

Self-efficacy was assessed in 5 [32,35,38,41,45] out of the 15 (33.3%) studies [32-46] using different validated instruments. In this regard, general self-efficacy was measured using the General Self-Efficacy Scale (GSES) in 1 study [45]; cancer-specific self-efficacy was measured in 2 studies through the Stanford Emotional Self-Efficacy Scale – Cancer (SESES-C) [32] and the Stanford Inventory of Cancer Patient Adjustment (SICPA) [35]; self-efficacy for symptom management was assessed with the Patient-Reported Outcomes Measurement Information System (PROMIS) Self-Efficacy for Managing Symptoms (SEMS) [41] and the Self-Efficacy for Managing Chronic Disease Scale (SEMCD) in another study [38]. These tools were designed to assess participants’ confidence in managing various aspects of their health, such as fatigue, emotional distress, and treatment-related challenges. Regarding effectiveness, the direction of effect favored the intervention in only 1 of the 5 trials. In that study, the professionally moderated Breast Cancer e-Support intervention improved cancer-related self-efficacy at 3 months, but the effect was not maintained at 6 months [35]. The remaining studies showed no clear between-group benefit. The certainty of evidence for self-efficacy was very low (Multimedia Appendix 3).

### Anxiety, Depression, and Emotional Distress

Anxiety was assessed in 11 (73.3%) out of the total selected studies [33-35,37-39,42-46] and was most commonly measured with the HADS-A, used in 6 studies [34,35,39,44-46]. Additionally, the Self-Rating Anxiety Scale (SAS) was used in 1 study [37], the Depression Anxiety and Stress Scale (DASS-21) in 2 studies [38,43], the State-Trait Anxiety Inventory in 1 study [33], and the anxiety item of the Patient-Reported Outcomes version of the Common Terminology Criteria for Adverse Events in 1 study [42]. Depression, on the other hand, was assessed in 8 (53.3%) studies [34,35,37-39,43,45,46], using the HADS-D in 5 studies [34,35,39,45,46], the DASS-21 [38,43], and the SDS in 1 study [37]. Emotional distress was assessed in 6 (40%) studies using the Profile of Mood States (POMS) and Hopkins Symptom Checklist-25 (HSCL-25) [32], the MD Anderson Symptom Inventory [36], the Distress Thermometer [37,44], and the stress subscale of the DASS-21 [38,43]. The direction of effect favored the intervention in 5 [36,37,39,44,46] of 13 studies [32-39,42-46]. Four of the 12 RCTs favored the intervention, whereas the remaining RCTs showed no clear benefit, effects favoring the comparator, or mixed findings. In the quasi-experimental study, anxiety favored the intervention group at 3 months, although no clear within-group reduction was observed and depression did not differ between groups [46]. Overall, the findings were heterogeneous and generally limited to short-term follow-up. The certainty of the randomized evidence was low.

### Acceptability

Regarding acceptability, only 4 studies reported information on this [38,43-45]. Two studies reported good acceptability among participants in the intervention groups [38,43]. Satisfaction was assessed in 2 studies [44,45]; however, one found no statistically significant between-group difference in perioperative care satisfaction [45]. Because acceptability and satisfaction were inconsistently defined and measured, no overall conclusion regarding intervention acceptability could be established.

### Retention Rate

Regarding attrition, the lowest retention rate was 68.6% [43], whereas the highest rate was 100% [46].

### Quality Appraisal

**Figure 2.**
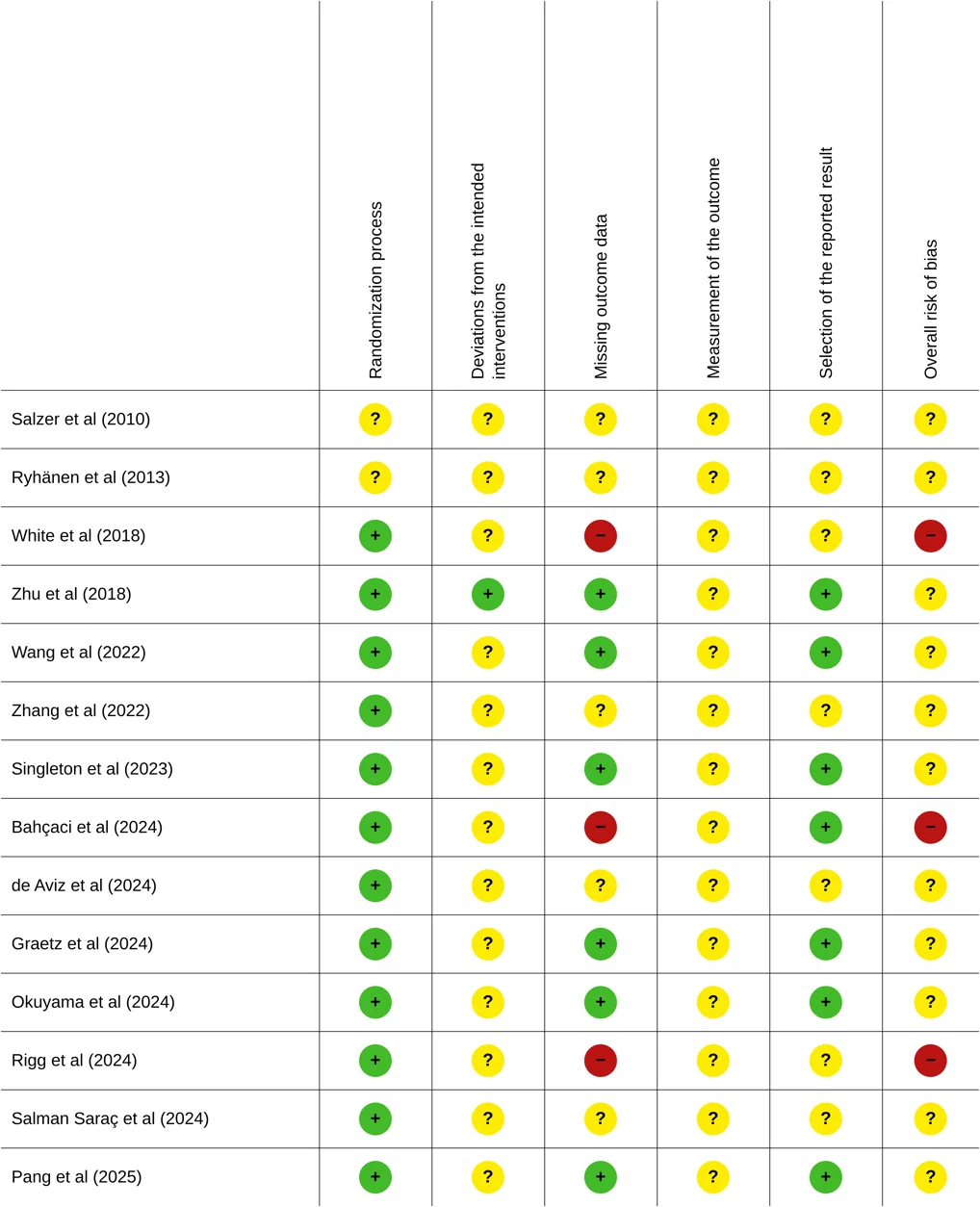
Risk of bias of the randomized controlled trials (RoB 2) [32-45].

The RoB 2 assessment included only the 14 RCTs and is presented in Figure 2. No trial was considered to be at low overall risk of bias. Three trials were judged to be at high overall risk of bias, mainly because of missing outcome data [34,39,43], including a pilot trial with a 34% loss to follow-up and differences between those who completed the study and those who did not [43]. The remaining 11 trials raised some concerns. Concerns were identified regarding outcome measurement because the main outcomes were subjective and self-reported by participants who were aware of their allocation to the intervention, and this knowledge could have influenced their reports. Across domains, most studies showed an adequate randomization process (D1), with allocation concealment unclear in a few; deviations from the intended interventions (D2) were generally limited but unblinded; missing outcome data (D3) was the main driver of high risk, with insufficient retention or incomplete handling of attrition in several trials; and selection of the reported result (D5) raised few concerns, while outcome measurement (D4) was limited throughout by self-reported, participant-aware assessment.

The quasi-experimental study was assessed separately using ROBINS-I and was judged to have a serious overall risk of bias [46]. The main concerns arose from the nonconcurrent sequential allocation of the control and intervention groups, the absence of statistical adjustment for potential confounding, and the measurement of subjective self-reported outcomes in participants who were aware of their intervention assignment. Risk was judged to be low for intervention classification and missing data, moderate for participant selection, deviations from intended interventions, and selection of the reported result, and serious for confounding and outcome measurement.

### Certainty of the Evidence

The certainty of the evidence was rated for each outcome using the GRADE approach [26,27]. Starting from high certainty, the evidence was downgraded for risk of bias and inconsistency across studies. No randomized trial was judged to be at low overall risk of bias, and 3 were at high risk. Certainty was low for QoL and for anxiety, depression, and emotional distress, and very low for self-efficacy, for which the evidence base was small and the instruments were heterogeneous. These ratings indicate that the short-term benefits observed should be interpreted with caution. Further details on the certainty assessments are provided in Multimedia Appendix 3.

## Discussion

### General Overview

Considering the advancement of digital health interventions in their various forms, it is essential to conduct critical reviews of the existing evidence, focusing on studies with high-quality scientific designs. This systematic review aimed to analyze the published research on digital health interventions in women with breast cancer and critically evaluate the effectiveness of these interventions. Specifically, it assessed the effects of digital health interventions on QoL and other psychological and behavioral variables, such as self-efficacy, anxiety, and depression.

### Principal Findings

#### QoL

With respect to QoL—the primary outcome of this review—the direction of effect favored the intervention in 8 [34-37,39,40,43,46] of the 15 studies [32-46], although the certainty of this evidence was low. Positive results were observed primarily in interventions such as digitally delivered mindfulness [36], VR-assisted psychotherapy [37] or via a website [43], telerehabilitation or guided relaxation [39,40], and a tailored mobile health (mHealth) physical activity intervention grounded in social cognitive theory (SCT), self-efficacy theory, and the theory of planned behavior (TPB) [46]. These interventions incorporated active therapeutic or behavioral components and, in most cases, some degree of professional guidance or personalized support. However, in the quasi-experimental study, total QoL did not significantly improve within the intervention group, and the between-group difference mainly reflected deterioration in the control group during chemotherapy, suggesting a relative preservation of QoL rather than a clear improvement attributable to the intervention; therefore, these findings should be interpreted cautiously given the nonrandomized design and serious overall risk of bias [46]. By contrast, passive interventions generally showed no clear between-group differences in QoL, with the exception of those incorporating health care team support through alert-triggered calls or professional moderation [35]. This trend has been corroborated by recent meta-analyses of digital interventions, which demonstrate that clinician-led support yields superior outcomes compared to stand-alone passive interventions [47-49].

Furthermore, improvements in QoL were primarily short-term. Follow-up beyond 6 months was uncommon, although 3 studies included assessments at or around 12 months [32,33,41], a duration generally recommended as a baseline for assessing the durability of effects and avoiding the overestimation of results [50,51]. This lack of longitudinal data, compounded by the complexity of the oncological process, where symptoms arising from both the cancer and its treatments converge, alongside factors such as digital fatigue due to the loss of the “novelty effect,” the absence of continuous human interaction, and the limited integration of digital interventions into long-term daily routines, calls into question the long-term clinical relevance of these interventions [52-54]. These observations suggest that sustaining benefits beyond the initial intensive phase may require ongoing human support or periodic reinforcement rather than one-off, self-guided exposure. However, given the low certainty of the current evidence and the absence of trials directly comparing such strategies, the optimal format, intensity, and timing of reinforcement remain undetermined and should be examined in future research before specific recommendations can be made.

#### Self-Efficacy

Across the selected studies, few effects were reported. Only one article showed, among its results, improvements in self-efficacy through the use of a supportive mobile app, which were observed particularly at 3 months of using said application; however, such effects were not maintained over time, with no significant improvements at the 6-month assessment [35]. In all the other interventions conducted, those that incorporated SMS delivery [38], an unmoderated peer listserve via email [32], mobile apps for symptom monitoring [41], and perioperative support [45], no differences between groups were evidenced. The foregoing, according to recent literature [55,56], could originate in the conception of the intervention design, since self-efficacy, in accordance with SCT, is sustained by mastery experiences, gradual achievements, and continuous feedback [57]; therefore, the limited active support of the intervention may affect the confidence acquired by patients, which could ultimately explain both the null effects and, at the same time, their limited persistence over time.

#### Anxiety, Depression, and Emotional Distress

Of the total number of articles, 13 evaluated anxiety, depression, or emotional distress, yielding mixed results [32-39,42-46]; the direction of effect favored the intervention in a minority of them (5/13) [36,37,39,44,46], and the certainty of this evidence was low. In this regard, interventions that incorporated telerehabilitation based on relaxation exercises delivered through messaging apps [39] and psychotherapy via VR [37] showed significant reductions in anxiety, depression, or distress; while mindfulness delivered by messaging generated initial changes in emotional distress; however, the results did not persist at the 1-month follow-up [36]. Favorable effects on anxiety or distress were also observed with an informative mobile app [44] and a theory-based tailored mHealth physical activity intervention [46]; however, in the latter study, anxiety did not significantly decrease within the intervention group, and no between-group difference was observed for depression. By contrast, informational or self-guided interventions delivered via the internet or a website [33-35], empowerment message delivery [38], symptom monitoring [42], and perioperative support [45] did not show differences compared with the control group, while psychological distress and mood favored the control group in one study [32] and another study reported poorer outcomes in the intervention group [43], although its small sample size limits conclusions regarding efficacy.

The foregoing could be explained by the degree of immersion and interactivity of the digital tool used, insofar as psychotherapy via VR provides a multisensory stimulus that could be capable of interrupting the anxiety cycle [58]. In this sense, these findings are consistent with previous evidence [59], as well as with relaxation videos grounded in Mayer’s modality principle [60], whereby the induction of calm through videos replaces the cognitive load associated with reading with an immersive experience that facilitates emotional regulation. In contrast, informational and monitoring platforms may be insufficient to address the affective component, while, as observed in the mindfulness intervention, one-directional support via messaging yields benefits that are not maintained in the long term, because digital mental health depends on proactive human support [61,62]. Across outcomes, the level of human support emerged as a key interpretive axis: guided interventions—those embedding professional facilitation or interaction—were more consistently associated with favorable effects than unguided, self-directed formats, in line with evidence that human guidance enhances the impact of digital health interventions [63,64].

### Strengths and Limitations

The main strength of this study lies in the predominance of RCTs, which represented 14 [32-45] of the 15 included studies [32-46], together with the inclusion of one eligible controlled quasi-experimental study in accordance with the predefined eligibility criteria. The inclusion of articles in English, Spanish, and Portuguese also expanded the knowledge base. The findings allow for a systematization of the current state of the art regarding the implementation of different types of digital health tools used to support women with breast cancer and their effects compared with standard care. Furthermore, this review underscores the need for the development of more rigorous randomized trials with greater statistical power in breast cancer populations throughout the entire oncological process.

Despite the contributions of this review, several limitations must be acknowledged. First, there was substantial heterogeneity in the measurement tools used to assess outcomes, which generated discrepancies in how QoL and other psychological variables were operationalized. Additionally, there was wide variability in the types of digital health interventions used, including mobile apps, text messaging, web-based platforms, and even VR devices. This variability extended to the technological formats, content, duration, level of human support, and timing of the interventions, limiting direct comparison across studies and reducing the ability to draw generalizable conclusions. Risk of bias was also a concern; no RCT was judged to be at low overall risk, and 3 trials were judged to be at high risk, primarily because of missing outcome data. The remaining trials raised some concerns, including concerns related to subjective, self-reported outcomes assessed by participants who were aware of their intervention assignment. The quasi-experimental study was judged to have a serious overall risk of bias and was therefore interpreted cautiously.

Finally, the search did not include MEDLINE or a systematic search of clinical trial registries or gray literature, which may have limited the retrieval of relevant or unpublished studies. Furthermore, the comparison between guided and unguided interventions was exploratory and based on indirect comparisons rather than a formal subgroup analysis.

### Conclusion

Digital health interventions for women with breast cancer may provide partial and mostly short-term benefits in some domains of health-related QoL and emotional well-being. Favorable effects were more frequently observed in interventions containing active therapeutic components or professional support; however, this pattern was based on indirect comparisons across heterogeneous studies. Nevertheless, several caveats temper these findings. Acceptability was formally assessed in only a few studies, so it cannot yet be considered established; effects on self-efficacy and on anxiety, depression, and emotional distress were limited and inconsistent, and were generally not maintained over time. Moreover, the certainty of the evidence was low to very low because of concerns regarding risk of bias and inconsistency across studies. Taken together, these findings should be interpreted with caution: digital health interventions are a promising adjunct in this population, but current evidence does not yet support strong or generalizable conclusions. Future research should prioritize methodologically rigorous, adequately powered trials with longer follow-up, standardized outcomes, and attention to adherence and the role of human support.

## Supplementary material

10.2196/96858 Multimedia Appendix 1 Search strategy.

10.2196/96858 Multimedia Appendix 2 Reports excluded.

10.2196/96858 Multimedia Appendix 3 Certainty of the evidence.

10.2196/96858 Checklist 1 PRISMA checklist.

## Abbreviations

CBT: cognitive behavioral therapy
DASS-21: Depression Anxiety and Stress Scale
EORTC QLQ-BR23: European Organisation for Research and Treatment of Cancer Quality of Life Questionnaire–Breast Cancer Module 23
EORTC QLQ-C30: European Organisation for Research and Treatment of Cancer Quality of Life Questionnaire–Core 30
GRADE: Grading of Recommendations Assessment, Development and Evaluation
GSES: General Self-Efficacy Scale
HSCL-25: Hopkins Symptom Checklist-25
mHealth: mobile health
PICOC: Population, Intervention, Comparison, Outcomes, and Context
POMS: Profile of Mood States
PRISMA: Preferred Reporting Items for Systematic Reviews and Meta-Analyses
PROMIS: Patient-Reported Outcomes Measurement Information System
QoL: quality of life
RCT: randomized controlled trial
RoB 2: Cochrane Risk of Bias tool
ROBINS-I: Risk of Bias in Non-randomized Studies of Interventions
SAS: Self-Rating Anxiety Scale
SCT: social cognitive theory
SEMCD: Self-Efficacy for Managing Chronic Disease Scale
SEMS: Self-Efficacy for Managing Symptoms
SESES-C: Stanford Emotional Self-Efficacy Scale – Cancer
SF-12: Short Form 12-Item Health Survey
SICPA: Stanford Inventory of Cancer Patient Adjustment
STAI: State-Trait Anxiety Inventory
SWiM: Synthesis Without Meta-analysis
TPB: theory of planned behavior
VR: virtual reality
WHO: World Health Organization
