# Supplementary material for: Digital Health Interventions and Psychosocial Outcomes in Women With Breast Cancer: Systematic Review
Source: JMIR Cancer. 2026 Aug 21;12:e96858. doi: 10.2196/96858 (PMC13496392; doi:10.2196/96858)
Supplement: Supplementary file 1 [file cancer-v12-e96858-s001.docx]

**Multimedia Appendix 1. Search strategy**

| **Database** | **Search** | **Total** |
| --- | --- | --- |
| APA PsycINFO (ProQuest) | 1: tiab(Digital Health*)  2:tiab(Digital Health Technolog*)  3: tiab(Virtual reality*)  4: tiab(mhealth*)  5: tiab(mobile health*)  6: tiab(Internet-Based Intervention*)  7: tiab(Internet Based Intervention*)  8: tiab(telemedicine*)  9: tiab(telerehabilitation*)  10: ab(digital*)  11:tiab(electronic health*) OR tiab(ehealth*) OR tiab(electronic*)  12: S1 OR S2 OR S3 OR S4 OR S5 OR S6 OR S7 OR S8 OR S9 OR S10 OR S11  13: tiab(Breast Neoplasm*)  14: tiab(Breast Tumor*)  15: tiab(Breast Cancer*)  16: ti(breast*)  17: S13 OR S14 OR S15 OR S16  18: tiab(QoL)  19: tiab(QLQ)  20: tiab(HRQOL)  21: tiab(quality of life*) OR ab(quality-of-life*)  22: tiab(health related quality of life*) OR tiab(health-related quality of life)  23: S18 OR S19 OR S20 OR S21 OR S22  24: tiab(Self efficacy*) OR tiab(Self-efficacy*)  25: tiab(anxiet*) OR tiab(anxiou*) OR tiab(angst*)  26: tiab(depress*) OR tiab(emotional depress*)  27: tiab(mindful*) OR tiab(mindfulness-based*)  28: S24 OR S25 OR S26 OR S27  29: S12 AND S17 AND S23 AND S28 | 46 |
| Web of Science Core Collection (CLARIVATE) Science Citation Index Expanded, Social Science Citation Index, Conference Proceedings Citation Index – Science, Conference Proceedings Citation Index - Social Science and Humanities, Emerging Sources Citation Index. | 1:TI=(Digital Health*) OR AB=(Digital Health*)  2:TI=(Internet Based Intervention*) OR AB=(Internet Based Intervention*)  3: TI=(Virtual reality*) OR AB=(Virtual reality*)  4:TI=(mhealth*) OR TI=(mobile health*) OR AB=(mhealth*) OR AB=(mobile health*)  5:TI=(Internet-Based Intervention*) OR AB=(Internet-Based Intervention*) OR TI= (Internet Based Intervention*) OR AB=(Internet Based Intervention*)  6:TI=(Telemedicine*) OR AB= (Telemedicine*) OR TI=(Telerehabilitation*) OR AB=(Telerehabilitation*)  7:TI=(Digital intervention*) OR AB=(Digital intervention*)  8: TI= (eHealth*) OR AB=(eHealth*)  9: TI=(Breast Neoplasm*) OR AB=(Breast Neoplasm)  10: TI=(Breast Tumor*) OR AB= (Breast Tumor*)  11: TI= (Breast Cancer*) OR AB=(Breast cancer*)  12: TI=(QoL) OR AB=(QoL)  13: TI=(QLQ) OR AB=(QLQ)  14: TI=(HRQOL) OR AB=(HRQOL)  15: AB=(Self efficacy) OR AB=(Self-efficacy)  16: TI=(Quality of Life) OR AB=(Quality of Life)  17: AB=(anxiet*) OR AB=(anxiou*) OR AB=(angst) OR AB=(depress*) OR AB=(Emotional Depression)  18: #1 OR #2 OR #3 OR #4 OR #5 OR #6 OR #7 OR #8  19: #9 OR #10 OR #11  20: #12 OR #13 OR #14 OR #16  21: AB=(mindfulness*) OR AB=(mindfulness-based*)  22: #18 AND #19 AND #20  23: #15 OR #17 OR #21  24: #22 AND #23 | 143 |
| LILACS (Virtual Health Library) | ( (digital health*) OR (digital*) OR (digital health technolog*) OR (health technolog*, digital) OR (internet based intervention*) OR (internet intervention*) OR (internet*) OR (web-based intervention*) OR (online intervention*) OR (intervention*, internet-based) OR (intervention*, internet) OR (online*) OR (mhealth*) OR (mobile health) OR (ehealth*) OR (electronic health*) OR (telehealth*) OR (telemedicine*) OR (telerehabilitation*) OR (virtual realit*) OR (remote consultation) OR (application*) ) AND ( (breast neoplasm*) OR (neoplasm*, breast) OR (breast tumor*) OR (breast cancer*) OR (cancer of breast) OR (cancer of the breast) ) AND ( (quality of life) OR (life quality) OR (health-related quality of life) OR (health related quality of life) OR (hrqol) OR (qol) OR (qlq) OR (self efficacy*) OR (self-efficacy*) OR (angst*) OR (anxiet*) OR (anxiou*) OR (depress*) OR (emotional depress*) OR (mindful*) OR (mindfulness-based*) ) AND instance:"lilacsplus" | 81 |
| SciELO | ( (digital health*) OR (digital*) OR (digital health technolog*) OR (health technolog*, digital) OR (internet based intervention*) OR (internet intervention*) OR (internet*) OR (web-based intervention*) OR (online intervention*) OR (intervention*, internet-based) OR (intervention*, internet) OR (online*) OR (mhealth*) OR (mobile health*) OR (ehealth*) OR (electronic health*) OR (telehealth*) OR (telemedicine*) OR (telerehabilitation*) OR (virtual realit*) OR (remote consultation) OR (application*) ) AND ( (breast neoplasm*) OR (breast tumor*) OR (breast cancer*) OR (cancer of breast) OR (cancer of the breast) ) AND ( (quality of life) OR (life quality) OR (health-related quality of life) OR (health related quality of life) OR (hrqol) OR (qol) OR (qlq) OR (self efficacy*) OR (self-efficacy*) OR (angst*) OR (anxiet*) OR (anxiou*) OR (depress*) OR (emotional depress*) OR (mindfulness*) ) | 6 |
| Scopus | TITLE-ABS-KEY ( ( digital health* ) OR ( digital* ) OR ( digital health technolog* ) OR ( health technolog* digital ) OR ( internet based intervention* ) OR ( internet intervention* ) OR ( internet* ) OR ( web-based intervention* ) OR ( online intervention* ) OR ( intervention* internet-based ) OR ( intervention* internet ) OR ( online* ) OR ( mhealth* ) OR ( mobile health ) OR ( ehealth* ) OR ( electronic health* ) OR ( telehealth* ) OR ( telemedicine* ) OR ( telerehabilitation* ) OR ( virtual realit* ) OR ( remote consultation ) OR ( application* ) ) AND TITLE-ABS-KEY ( Breast Neoplasm* OR Breast Tumor* OR Breast Cancer* OR Breast* ) AND TITLE-ABS-KEY ( ( quality of life* ) OR ( life quality ) OR ( health-related quality of life* ) OR ( health related quality of life* ) OR "hrqol" OR "qol" OR "qlq" OR ( self efficacy* ) OR ( self-efficacy* ) OR angst* OR anxiet* OR anxiou* OR depress* OR ( emotional depress* ) OR mindful* OR mindfulness-based* ) | 132 |
| Total retrieved | | 408 |
| Possible duplicates | | 122 |
| Auto resolve >95% | | 79 |
| Manual resolve | | 43 |
| Final number for screening | | 286 |
