# Supplementary material for: Digital Health Interventions and Psychosocial Outcomes in Women With Breast Cancer: Systematic Review
Source: JMIR Cancer. 2026 Aug 21;12:e96858. doi: 10.2196/96858 (PMC13496392; doi:10.2196/96858)
Supplement: Supplementary file 3 [file cancer-v12-e96858-s003.docx]

**Multimedia Appendix 3. Certainty of the Evidence**

| **Outcome** | **Studies (participants)** | **Initial certainty** | **Factors reducing certainty** | **Certainty (GRADE)** | **Interpretation** |
| --- | --- | --- | --- | --- | --- |
| Quality of life (primary) | 14 RCTs | High (RCTs) | Risk of bias (−1): no trial was at low overall risk; 3 were at high risk, mainly because of missing outcome data. Inconsistency (−1): favourable direction in 7/14, short-term and modality-dependent. | Low | Digital interventions may improve some QoL domains, mainly in the short term; evidence is uncertain. |
| Anxiety / depression / emotional distress | 12 RCTs | High (RCTs) | Risk of bias (−1): concerns arose mainly from missing outcome data and self-reported outcomes. Inconsistency (−1): favourable direction in only 4/12; heterogeneous constructs and instruments (HADS, STAI, SAS, SDS, DASS-21, DT, POMS, HSCL-25, MDASI, and PRO-CTCAE). Imprecision: small samples in several trials (no additional downgrade). | Low | May reduce emotional distress when interventions include active components or human support; evidence is inconsistent. |
| Self-efficacy | 5 RCTs | High (RCTs) | Risk of bias (−1): all 5 trials raised at least some concerns, mainly because outcomes were self-reported. Inconsistency (−1): positive effect in only 1/5 and not sustained. Imprecision / indirectness (−1): small evidence base and instruments measuring different constructs (general, cancer-specific, and symptom-management self-efficacy). | Very low | It is very uncertain whether interventions improve self-efficacy; the single positive effect was not maintained over time. |

The GRADE certainty assessments were based on the randomized evidence, and the risk-of-bias domain was informed by the RoB 2 assessment of the randomized controlled trials. The quasi-experimental study was included in the narrative synthesis but was not incorporated into the GRADE certainty assessments. Publication bias could not be formally assessed because no meta-analysis or funnel plot was performed and was therefore reported as not assessed.
